# Gastric Adenocarcinoma Incidentally Detected by PET/CT with PSMA Ligands

**DOI:** 10.3390/diagnostics15010101

**Published:** 2025-01-03

**Authors:** Cesare Michele Iacovitti, Barbara Muoio, Marco Cuzzocrea, Gaetano Paone, Giorgio Treglia

**Affiliations:** 1 Division of Nuclear Medicine, Imaging Institute of Southern Switzerland, Ente Ospedaliero Cantonale, 6500 Bellinzona, Switzerland; cesaremichele.iacovitti@eoc.ch (C.M.I.); marco.cuzzocrea@eoc.ch (M.C.); gaetano.paone@eoc.ch (G.P.); 2 Division of Medical Oncology, Oncology Institute of Southern Switzerland, Ente Ospedaliero Cantonale, 6500 Bellinzona, Switzerland; barbara.muoio@eoc.ch; 3 Faculty of Biomedical Sciences, Università della Svizzera Italiana, 6900 Lugano, Switzerland; 4 Faculty of Biology and Medicine, University of Lausanne, 1015 Lausanne, Switzerland

**Keywords:** PET, positron-emission tomography, nuclear medicine, hybrid imaging, PSMA, gastric cancer

## Abstract

Here, we describe the case of a 74-year-old male patient with a high-risk prostate carcinoma who underwent positron-emission tomography/computed tomography (PET/CT) with [^68^Ga]Ga-prostate-specific membrane antigen ([^68^Ga]Ga-PSMA-11) for staging. [^68^Ga]Ga-PSMA-11 PET/CT detected an extensive area of increased tracer uptake at the prostatic level, involving both lobes. Additionally, a rounded lesion approximately 4 cm in diameter was identified in the celiac region adjacent to the stomach, exhibiting moderate tracer uptake. Based on these imaging findings, the patient underwent radiation therapy applied to the prostate and pelvis and a biopsy of the suspected lesion adjacent to the stomach, which was positive for Siewert type III gastroesophageal junction adenocarcinoma (HER2-negative, PDL-1 60%). This case demonstrates the importance of not overlooking incidental tracer uptakes in PSMA PET/CT imaging in the stomach, as they could represent neoplastic lesions.

**Figure 1:**
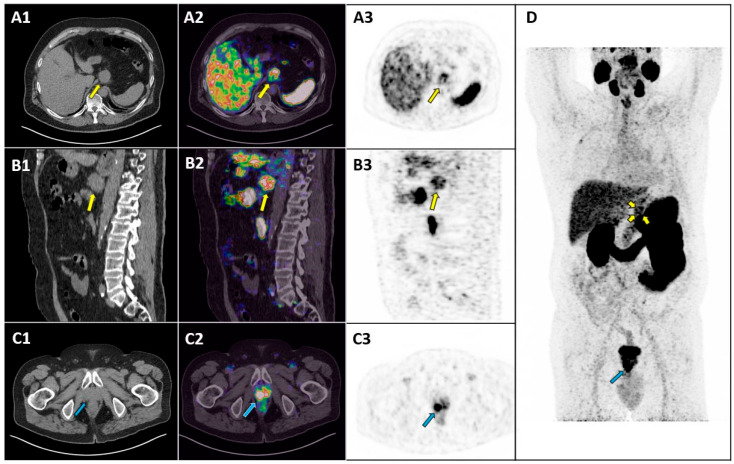
A 74-year-old male patient with a high-risk prostate carcinoma underwent positron-emission tomography/computed tomography (PET/CT) with [^68^Ga]Ga-prostate-specific membrane antigen ([^68^Ga]Ga-PSMA-11) for staging. PET/CT was performed 60 min after an intravenous injection of 250 MBq of [^68^Ga]Ga-PSMA-11. PET image analysis was performed using qualitative criteria: areas of increased tracer uptake compared to the background, excluding sites of physiological uptake, were considered abnormal. Axial CT image (**A1**), axial fused PET/CT image (**A2**), axial PET image (**A3**), sagittal CT image (**B1**), sagittal fused PET/CT image (**B2**) and sagittal PET image (**B3**) at gastric level revealed a rounded lesion (approximately 4 cm in diameter) adjacent to the stomach in the celiac region, exhibiting moderate tracer uptake (yellow arrows in (**A1**–**A3**,**B1**–**B3**,**D**); SUVmax: 9.7). This lesion appeared inseparable from the stomach wall. Axial CT image (**C1**), axial fused PET/CT image (**C2**) and axial PET image (**C3**) at the pelvic level revealed an extensive area of increased tracer uptake at the prostatic level involving both lobes (blue arrows in (**C1**–**C3**,**D**)). The maximum-intensity-projection (MIP) PET image (**D**) highlighted both the prostatic lesion (blue arrow) and the gastric lesion (yellow arrow), indicating abnormal tracer uptake at both sites. Upon physical examination after the PET/CT scan, the patient did not show any intestinal symptoms such as decreased appetite, indigestion, or abdominal distension. Based on these imaging findings, the patient underwent radiation therapy applied to the prostate and pelvis and a biopsy of the suspected lesion adjacent to the stomach. Endoscopic examination revealed a large vegetative lesion extending along the small curvature of the stomach from the cardia to the level of the gastric antrum. Histopathological examination demonstrated a Siewert type III gastroesophageal junction adenocarcinoma (HER2-negative, PDL-1 60%). PSMA PET/CT has an established role in prostate cancer imaging [1], but non-prostatic tumor lesions may exhibit PSMA overexpression and increased PSMA ligand uptake in PET/CT [2,3]. This case demonstrates the importance of not overlooking gastric incidental tracer uptakes in PET/CT with PSMA ligands, as they may represent a malignant tumor.

## Data Availability

The data presented in this article are available upon request from the corresponding author.
